# Comparing the Effects of Age, Sex, and Extremity Fracture Location on Continuous Intra-compartmental Pressure (ICP) Measurements in Trauma Patients

**DOI:** 10.7759/cureus.99300

**Published:** 2025-12-15

**Authors:** Justin Matta, Yasser C Bouklouch, William Obremskey, Ross Leighton, Mitchell Bernstein, Edward J Harvey

**Affiliations:** 1 Orthopaedic Surgery, McGill University, Montreal, CAN; 2 Orthopaedic Surgery, Vanderbilt University Medical Center, Nashville, USA; 3 Orthopaedic Surgery, Dalhousie University, Halifax, CAN

**Keywords:** #acute compartment syndrome, human physiology, major trauma, micro-electromechanical systems, orthopedic fractures

## Abstract

Background

Baseline data for extremity compartment pressure after trauma have only been looked at in small cohorts with historically inaccurate technology. Newer technology has enabled continuous, accurate pressure monitoring for the diagnosis of acute compartment syndrome (ACS). This study used data from prospective cohort trials with modern pressure sensors to examine baseline pressure values. Particularly, there was a comparison of the effects of age, sex, and fracture location on continuous trend behaviors in extremity trauma patients.

Methodology

Intracompartmental pressures (ICPs) from 129 non-ACS trauma patients with extremity fractures were examined. The trends in patients were analyzed with respect to age, sex, and anatomical fracture location.

Results

Younger patients exhibited higher mean ICPs compared to older patients at all time points. Both groups experienced the same rates of decline in pressure over time from trauma. Both older and younger patient groups experienced a steady linear decrease in pressure over the course of monitoring. The younger age group had a decrease of 0.202 mmHg per hour (y = 26.4 - 0.202x), and the older age group showed the same rate of decrease (y = 23.9 - 0.202x). Males and females initially had similar ICPs, but females showed a steeper decline over time, with the pressure in the female group decreasing at a mean rate of 0.303 mmHg/hour compared to 0.163 mmHg/hour in the male group. Tibia fractures were associated with a higher initial pressure and steeper declines in ICP compared to forearm fractures.

Conclusions

There are variances for continuous ICP measurements associated with age, sex, and fracture location in trauma patients who do not develop ACS. Continuous ICP monitoring offers a better understanding of pressure trends, allowing for more accurate and individualized assessments. Recognizing these trends is crucial in ACS assessment.

## Introduction

Fascial compartments that restrict muscle expansion represent a potential risk after trauma. Induced high intracompartmental pressure (ICP) can lead to tissue death [1,2]. Understanding pressure trends is crucial for the effective diagnosis and management of acute compartment syndrome (ACS) [3-5]. While the need for prompt surgical intervention has been well publicized, the clinical assessment of ACS remains subjective and lacks sensitivity and specificity [6-8]. At least two previous studies have looked at baseline pressures after trauma in patients who were thought to be low risk for ACS [9,10]. These studies, unfortunately, centered around single-point measurement with technology that is less than ideal [9,11,12]. Under normal physiological conditions, ICP fluctuates [3,13-16]. Several factors have been postulated to contribute to variations in ICP among individuals, specifically with regard to sex, age, and anatomical location, which, in turn, may influence baseline ICP patterns [3,7,17]. While various absolute pressure trigger points for surgery in ACS have been suggested [2,18-21], this literature was written before modern methods of measuring pressure [11-13].

Different muscle groups have been claimed to have different pressure responses [22-26]. Anatomical differences in the size and structure of muscle compartments vary between individuals, along with proportions of metabolically active muscle types, which may affect baseline compartmental pressures and their reactions to trauma and reperfusion. Recent studies have indicated notable clinical differences in incidence between males and females [7,27]. Males have a reported incidence rate of 7.3 per 100,000, while females have a rate of 0.7 per 100,000. Studies have shown that females are at lower risk than males in lower leg compartments, and studies of national databases support this finding [7,27,28]. Younger age has also been identified as one of the major risk factors for developing compartment syndrome following a tibial fracture [1,3,7,29]. The anterior compartment of the lower leg following a tibial fracture is also the most common site of ACS, presumably in part due to its anatomical constraints, the location of its neurovasculature, and the rigidity of the surrounding fascia [30-32]. The volar compartment of the forearm is the second most common site of ACS [33], but there is a difference in incidence after trauma between these two sites. Though hypothesized, the reasons for the disparity in incidence between the lower leg and forearm remain poorly understood.

Many variables, such as timing and severity of the injury, contribute to the complex and dynamic nature of compartmental pressures and highlight the need for individualized and more objective clinical assessment approaches. While the progressive adoption of continuous ICP monitoring is a more informative tool [3,4,29,34,35], expected values have yet to be determined. This study expands on the understanding of continuous trends among varying demographics using an accumulation of anonymized, continuous, accurate pressures collected during multiple prospective cohort trials. The hypothesis was that pressure trends in patients following extremity trauma may be different in males, younger individuals, and those sustaining a lower limb fracture.

## Materials and methods

Pressure data were captured using the My01 (Montréal, Canada) ICP monitoring device in prospective cohort trials. Anonymized pressure data files were supplied for this study. The study population comprised 139 patients. Patients who developed acute compartment syndrome were excluded (10 patients). This exclusion is meant to focus the analysis on non-pathological cases. Pressure tracings were obtained from ongoing prospective clinical trials [1-4]. All tracings and values from patients in two prospective cohort trials with tibial or forearm fractures were available for analysis. The pressure device was a micro-electrical machine system type with increased accuracy [12,36]. It was placed in either the anterior compartment of the leg or the volar wad of the forearm [4,17].

Data from devices that were inserted for less than one minute were excluded as trends could not be analyzed. Data were standardized, and graphs were produced using the GGPLOT2 library on R version 4.2.2 (R Foundation for Statistical Computing). Regression plots and linear equations (y = pressure in mmHg, x = time in hours) were generated using the ordinary least squares method. Curves included a generalized pressure curve based on the mean pressure at each time point and a quantile distribution for each time point.

## Results

**Figure 1:**
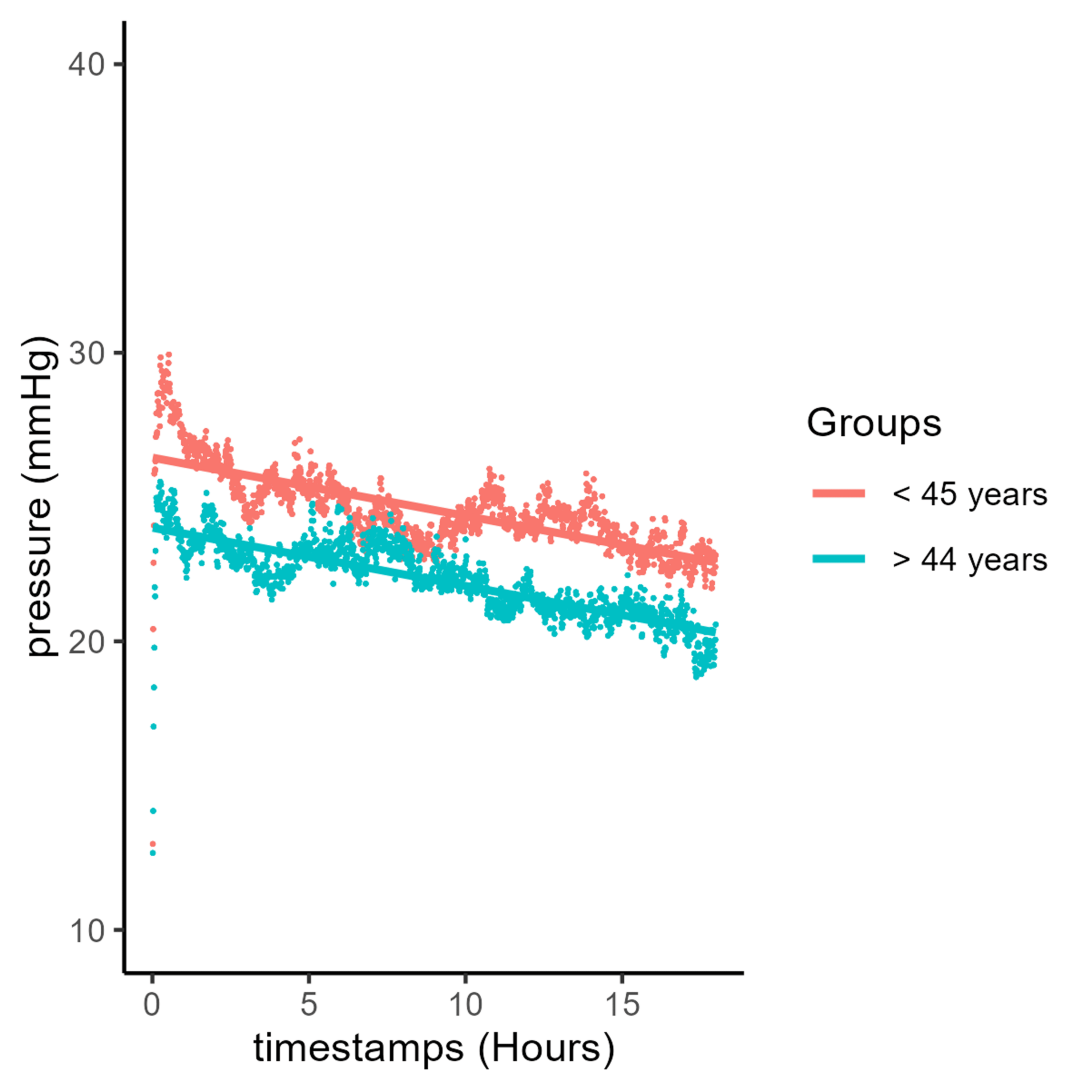
Mean muscle compartment pressure over time with a linear trend line depending on age group.

**Figure 2:**
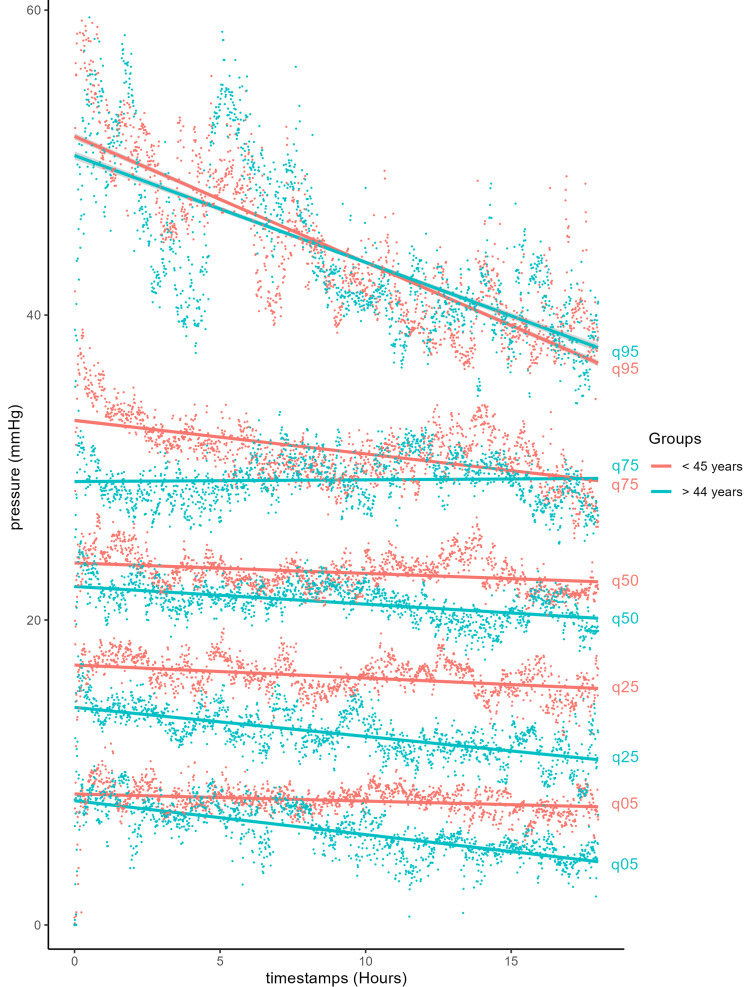
Quantile distribution of muscle compartment pressures in non-ACS patients by age group. ACS, acute compartment syndrome

The group was two-thirds male and one-third female (Table 1). The mean ages were 43 and 42 years in the tibial and forearm fracture groups, respectively. Following studies on the effect of age on muscle mass [37], we divided the cohort into two groups: a younger group (<45 years) and an older group (≥45 years). The mean ages for the younger and older groups were 31 and 58 years, respectively. We combined all pressure curves at each time point and calculated a mean value for every time point. Younger patients had higher mean compartment pressures than older patients at every time point during the monitoring period (Figure 1). There was some overlap in these pressures when examined by quantiles (Figure 2). Both the older and younger patient groups experienced a steady linear decrease in pressure over the 18-hour monitoring period. The younger group had an initial pressure of 26.4 mmHg with a decrease of 0.202 mmHg per hour (y = 26.4 − 0.202x), while the older group had an initial pressure of 23.9 mmHg with the same rate of decrease (y = 23.9 − 0.202x). At equal quantiles, pressures in older patients were lower by 2.5 mmHg compared with those of younger patients. Additionally, younger patients showed a very slow decline in pressure, except for the top 5% of patients, who experienced a rapid decrease (Figure 2).

**Table 1:** Description of study cohorts. ^a^*n* (%); mean ^b^Fisher's exact test for count data; Wilcoxon rank-sum test.

Characteristics	Tibia (*N* = 114)^a^	Forearm (*N* = 15)^a^	*P*-value^b^
Sex			0.8
F	37 (32%)	4 (27%)	
M	77 (68%)	11 (73%)	
Age (years)	44	39	0.4

**Figure 3:**
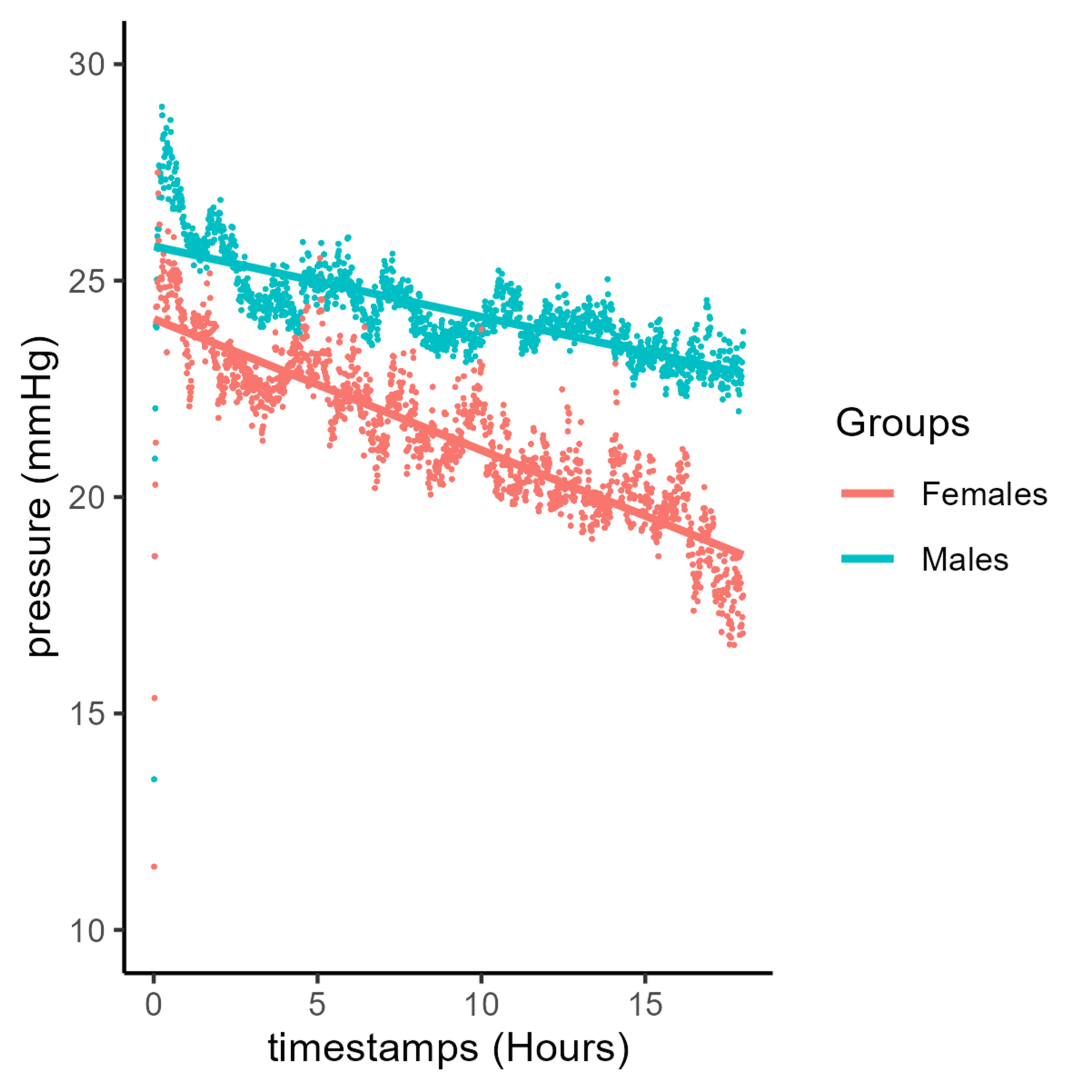
Mean muscle compartment pressure over time with a linear trend in males and females.

**Figure 4:**
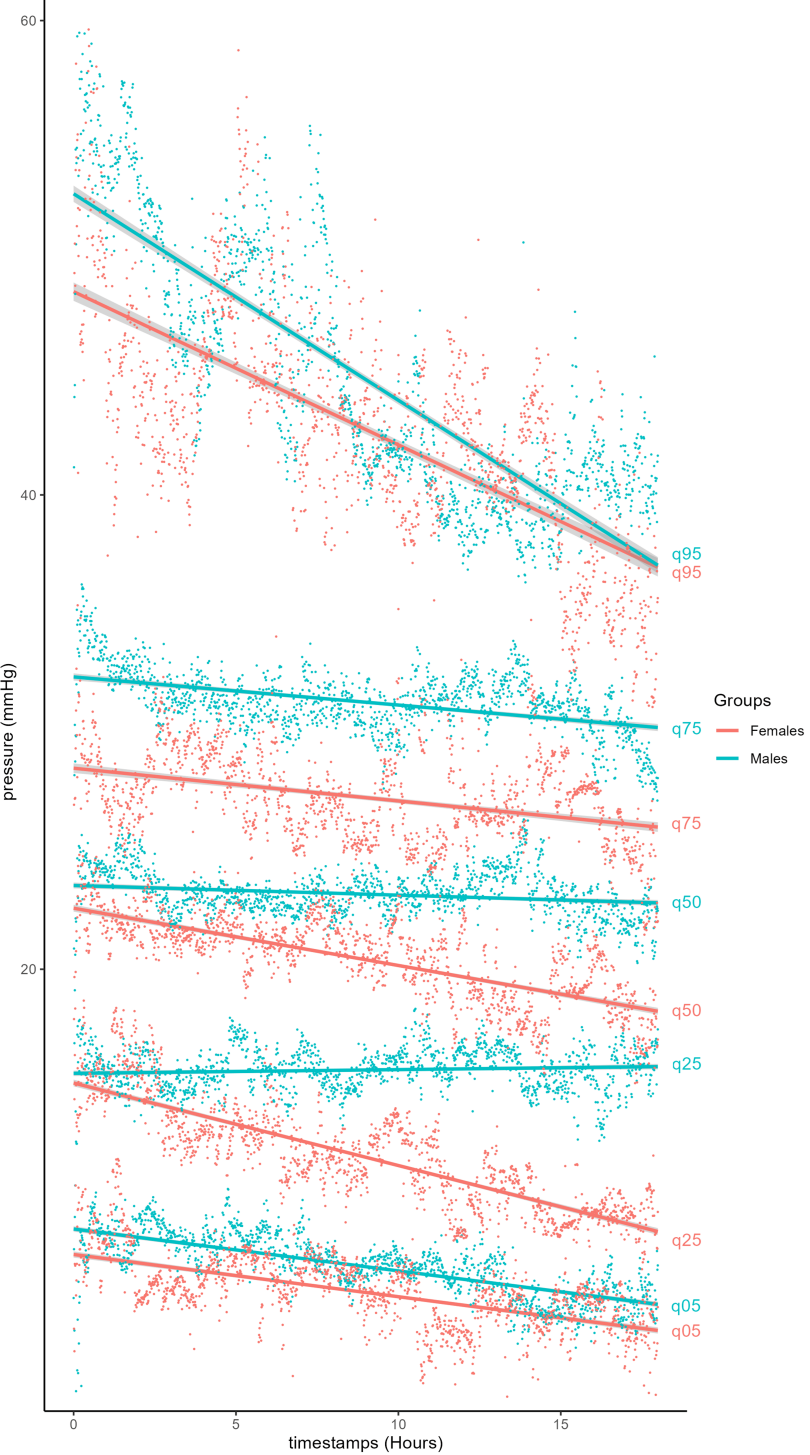
Quantile distribution of muscle compartment pressures in non-ACS patients depending on sex. ACS, acute compartment syndrome

Using the previous method, males and females had similar pressures at the time of insertion of the probe (Figure 3). However, the pressure in the female group decreased at a mean rate of 0.303 mmHg/hour (y = 24.1 - 0.303x), compared with 0.163 mmHg/hour (y = 25.8 - 0.163x) in the male group. The rate of decrease was linear among both groups. We proceeded to estimate the essential quantile thresholds (5th, 25th, 50th, 75th, and 95th percentiles) for each time point (Figure 4). At equivalent quantiles, males exhibited higher pressures than females throughout the monitoring period.

**Figure 5:**
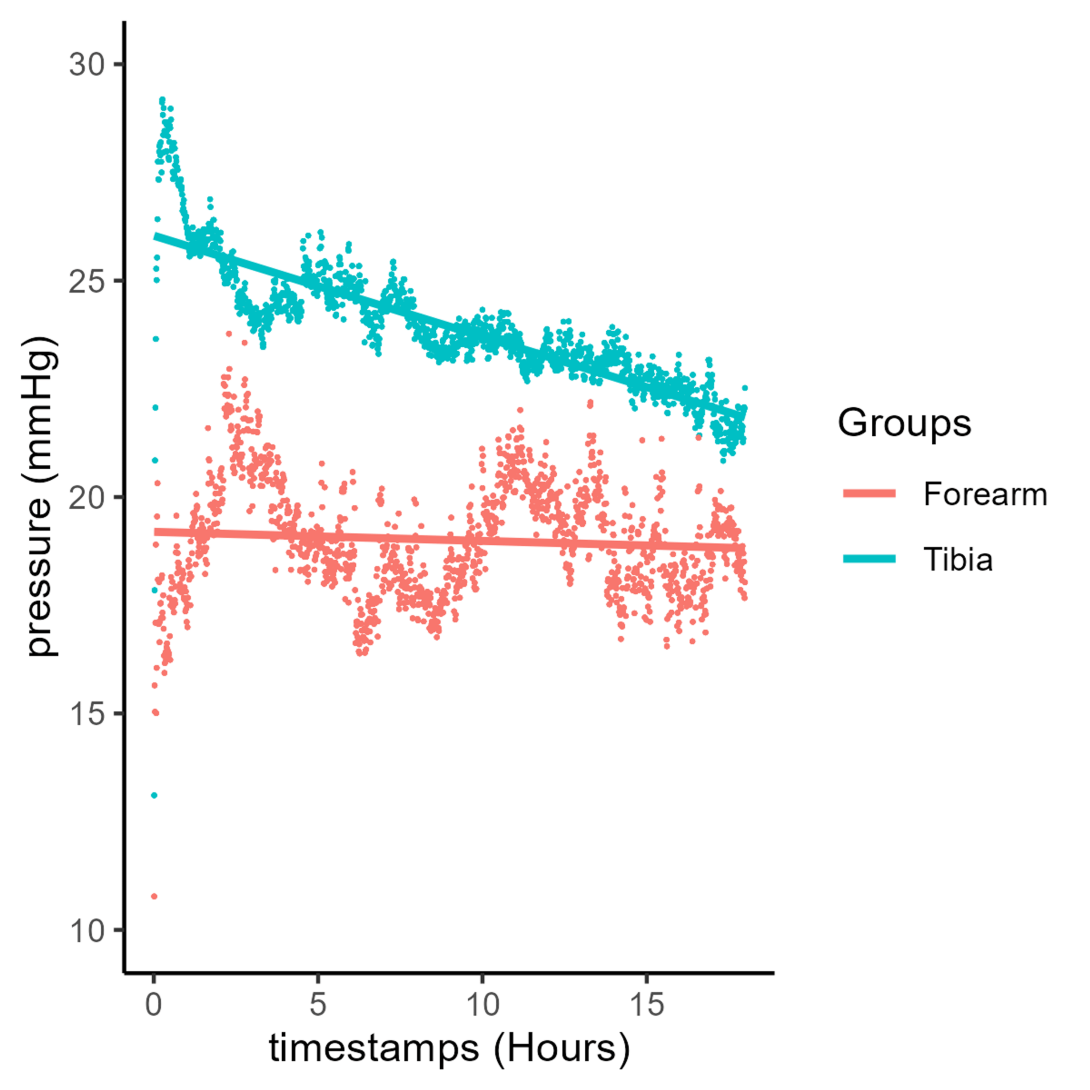
Mean muscle compartment pressure over time with linear trends in the forearm and tibia.

**Figure 6:**
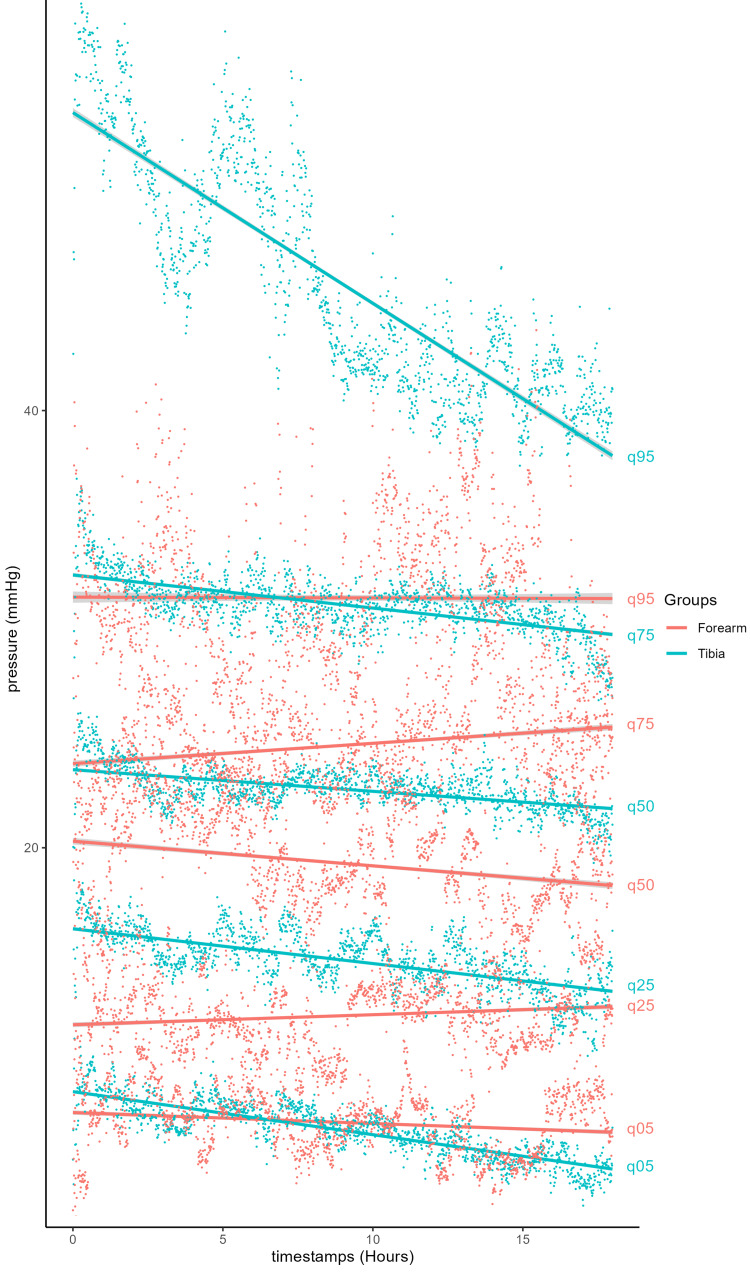
Quantile distribution of muscle compartment pressures in non-ACS patients with forearm and tibial fractures. ACS, acute compartment syndrome

Comparing anatomical regions, the average pressure recorded at the time of device insertion was 26 and 19.2 mmHg in tibia and forearm fractures, respectively. Tibial fractures tended to exhibit a steady decrease in mean pressure at 0.233 mmHg/hour (y = 26 - 0.233x), while forearm fractures showed a minimal decrease in pressure at 0.021 mmHg/hour (y = 19.2 - 0.021x) over a comparable monitoring period (Figure 5). This was confirmed by comparing the quantiles. At equivalent quantiles, forearm fractures showed little to no decrease in pressure, whereas tibial fractures exhibited a steady decline across all quantiles (Figure 6).

## Discussion

Despite reports of disparities in incidence based on sex, age, and anatomical site [7], objective diagnosis is crucial to reduce the risk of misdiagnosis, given that current clinical assessment is entirely subjective [28]. Without objective monitoring, these biases have led to an excess of prophylactic fasciotomy procedures in higher-risk populations [7], such as young males, while also delaying treatment in lower-risk populations, such as females and older individuals [38]. The current use of continuous, real-time monitoring of ICP has supported the diagnosis of ACS by combining clinical assessment with objective ICP measurements [4,28,34,35]. However, continuous pressure measurements and trends remain understudied, with only small cohorts reported [10,39]. This has hindered the incorporation of pressure trends, rather than single-point pressure values, into decision-making and diagnosis for ACS. This study aimed to improve the understanding of variations in continuous ICP among non-ACS patients differing in sex, age, and fracture location.

ICPs (Figure 1) in both younger and older patients decreased steadily over time, consistent with the fact that these patients did not develop ACS. Despite not developing ACS, both groups initially exhibited elevated ICPs, averaging 26.4 mmHg in the younger group and 23.9 mmHg in the older group, likely due to swelling, bleeding, and inflammation within the compartment. Initial (immediately post-monitor insertion) elevated ICP readings may also result from fluctuations caused by muscle spasm from trauma or device insertion, patient movement, splinting, or external palpation of the compartment, all of which can influence ICP values. This slightly higher initial pressure period (Figures 1-2) resolves within an hour, reinforcing the need to examine trends over time. Notably, however, a pressure difference is still evident throughout the observation period. The younger patient group maintained a consistent 2.5-mmHg higher pressure compared with the older group, while both groups followed the same rate of decline at 0.202 mmHg/hour. The continuous pressures measured are indicative of the pressure difference that can be expected in younger individuals, perhaps due to the larger and more abundant muscle, more restricted compartment, and varied proportions of muscle fibers [40,41]. A retrospective cohort study by McQueen was among the first to document youth as one of the main predictors of ACS following tibial fracture, which is supported by the continuous pressure measurements in this study [7,42].

Similar to age, sex also demonstrated notable differences in ICP (Figures 3-4). Although both groups began with relatively similar ICPs after trauma, the female group exhibited nearly double the rate of pressure decline compared with the male group, at 0.303 and 0.163 mmHg/hour, respectively. Due to the steeper decline and increasingly widening gap of 0.140 mmHg/hour, the female group had lower ICP compared to males by the final time points. This may reflect a higher baseline ICP in males. There may also be a difference in the level of trauma between males and females, as we did not have access to radiographs or images of the extremities. There may also be an as-yet poorly understood enhanced clearance of intracellular fluid from muscle in females. A simpler explanation could be lower muscle mass, resulting in a reduced response to trauma and allowing the muscles to clear the fluid that accumulates as part of the inflammatory cycle. Physiological differences between males and females are also reflected anatomically, with sex-specific hormones and differences in musculoskeletal gene expression leading to larger muscle mass and higher proportions of metabolically active and fast-twitch fibers in males compared to females [5-7,41]. These variances may influence the response within the compartments, potentially predisposing to ACS, while also reflecting sex-based differences in the rate of ICP decline observed in patients who do not develop ACS.

Interestingly, the ICP trendlines recorded following forearm and tibia fractures differed (Figures 5-6). Patients with tibial fractures had a higher mean pressure of 26 mmHg with a steeper decline of 0.233 mmHg/hour, whereas those with forearm fractures had a lower initial mean ICP of 19.2 mmHg, with an overall gradual decrease of 0.021 mmHg/hour (Figure 5) or values that remained statistically unchanged over time across quantiles (Figure 6). While the initial elevated pressure in the tibial group can be attributed to swelling and inflammation at the injury site, its gradual decline follows the trend expected in patients who do not develop ACS. Non-ACS patients with forearm fractures had initial ICP values 5.8 mmHg lower than those in the tibial group. This resulted in a difference in the rate of pressure decline of 0.212 mmHg/hour between the two groups. The forearm exhibits several anatomical and physiological differences compared with the lower leg, which is reflected in the distinct ICP trends observed. Compared with the lower leg’s four compartments, the forearm has three compartments with relatively smaller muscles, which may allow for greater accommodation of swelling. Over time, pressure variations may be more gradual when swelling occurs and less likely to reach the relative critical threshold characteristic of ACS. The relative vascular supply to the forearm is more robust than that of the anterior compartment of the lower leg [43], with increased blood flow and venous return potentially helping to mitigate pressure increases. Injuries leading to ACS in the forearm, such as fractures of the radius and ulna or crush injuries, are less common and generally less severe than those affecting the lower leg [7]. There were also far fewer forearm patients in the cohorts, which may simply reflect a statistical variance.

It is also important to note that the top 25% of patients in each group reached ICPs above 30 mmHg for part or all of the monitoring period, as shown in the quantile distribution graphs (Figures 2, 4, 6). While their numerical pressure values may have been among the highest in their demographic group, their pressure trends remained comparable in slope to those observed in the lower quantile trendlines. A limitation of this study is the small number of patients. All patients who had ACS were also removed from the cohort. Although none of the remaining patients developed ACS, many could have been susceptible to the recommended absolute pressure threshold for fasciotomy of 30 mmHg [2,6] if the observed trends had not occurred. This emphasizes the advantage of studying trendlines from continuous pressure monitoring as a more informative tool than single-point pressure measurements.

## Conclusions

This study provides insights into the variations of ICPs based on age, sex, and fracture location in trauma patients who do not develop ACS. These variances may be attributed to anatomical and physiological differences that influence compartmental pressures. These findings may also emphasize the utilization of pressure trendlines collected through continuous monitoring as a more informative and valuable tool compared to single-point measurements. Continuous ICP monitoring may provide a more comprehensive and individualized understanding of dynamic compartment pressure changes in patients following trauma. Considering variations in age, sex, and fracture location may improve understanding of the etiology and risk factors of ACS and enhance the accuracy of its diagnosis.
